# Processing of meats and cardiovascular risk: time to focus on preservatives

**DOI:** 10.1186/1741-7015-11-136

**Published:** 2013-05-23

**Authors:** Renata Micha, Georgios Michas, Martin Lajous, Dariush Mozaffarian

**Affiliations:** 1 Departments of Epidemiology and Nutrition, Harvard School of Public Health, 665 Huntington Ave Bldg 2-319, Boston, MA 02115, USA; 2 Division of Cardiovascular Medicine and Channing Division of Network Medicine, Department of Medicine, Brigham and Women’s Hospital and Harvard Medical School, Boston, MA 02115, USA; 3 Center for Research on Population Health, National Institute of Public Health, Cuernavaca, Mexico; 4 National Institute of Health and Medical Research (Inserm), Center for Research in Epidemiology and Population Health (CESP), and Gustave-Roussy Cancer Institute, U1018, Villejuif, France; 5 Department of Food Science and Technology, Unit of Human Nutrition, Agricultural University of Athens, Athens, Greece

**Keywords:** Review, Meat, Red meat, Processed meat, Cardiovascular diseases, Diabetes

## Abstract

Dietary guidelines emphasize selecting lean (low-fat) meats to reduce saturated fat and cholesterol, but growing evidence suggests that health effects may relate to other ingredients, such as sodium, heme iron, or L-carnitine. Understanding how meats influence health, and on which nutrients this relationship depends, is essential to advise consumer choices, set guidelines, and inform food reformulations. A recent study published in *BMC Medicine* involving 448,568 participants in 10 European countries, provides important evidence in this regard. After multivariate adjustment, intake of unprocessed red meat was not significantly associated with total or cause-specific mortality; conversely, intake of processed meat was associated with a 30% higher rate of cardiovascular disease (CVD) (per 50 g/day, relative risk 1.30, 95% confidence interval 1.17 to 1.45) and also higher cancer mortality. These findings are consistent with our previous meta-analysis, based on smaller studies, showing strong associations of processed meats, but not unprocessed meats, with CVD. Preservatives are the notable difference; the calculated blood-pressure effects of sodium differences (around 400% higher in processed meats) explain most of the observed higher risk. Although unprocessed red meats seem to be relatively neutral for CVD, healthier choices are available, including fish, nuts, legumes, fruits, and vegetables. Public-health guidance should prioritize avoidance of processed meats, including the low-fat deli meats currently marketed as healthy choices, and the food industry should substantially reduce sodium and other preservatives in processed meats.

See related research article here http://www.biomedcentral.com/1741-7015/11/63.

## Background

Eating red meat is commonly considered to be a major dietary risk for cardiovascular disease (CVD). Most of the focus has been on the saturated fat and cholesterol content, leading to public-health emphasis on selecting lean meats and moderating overall meat consumption [1], yet a growing body of evidence indicates that the story is not so simple. First, whether compared with the overall background diet or with carbohydrate consumption, overall intake of saturated fat is consistently unrelated to incidence of CVD [2-4]. Second, the health effects of red meat may be most strongly related to other ingredients, such as sodium or other preservatives present in processed meats [5], heme iron, which may increase the risk of diabetes [6-8], or L-carnitine, which may be metabolized by gut bacteria to pro-atherosclerotic compounds [9]. Understanding the relations of meat intake with major health outcomes, and on which key nutrients this relationship depends, is essential for guiding consumer choices, setting and prioritizing dietary guidelines, and informing food reformulations to reduce risks. The recent investigation by Rohrmann and colleagues [10] provides important evidence that helps further clarify these key issues.

## Discussion

The investigators evaluated how eating meat related to total and cause-specific mortality in the large European Prospective Investigation into Cancer (EPIC) cohort, including 448,568 participants in 23 participating centers across 10 European countries. Importantly, that study took care to separately evaluate unprocessed red meat, unprocessed poultry, and processed meats (including processed red meat and processed poultry). During an average follow-up of 12.7 years, 26,344 deaths occurred, comprising 5,556 due to CVD, 9,861 to cancer, 1,068 to respiratory disease, 715 to digestive tract diseases, and 9,144 to other causes. Notably, the authors appropriately accounted for potential effects of residual confounding (which would cause, in this case, overestimation of harm of meat intake) and random errors in diet assessment (which would cause underestimation of associations).

In calibrated and adjusted models for various lifestyle and dietary factors, consumption of unprocessed red meat was not significantly associated with CVD mortality (per 100 g/day, relative risk (RR) = 1.09, 95% confidence interval (CI) = 1.00 to 1.18); consumption of unprocessed poultry was associated with a non-significant trend toward lower risk (per 50 g/day, RR = 0.84, 95% CI = 0.69 to 1.03); and consumption of processed meat was associated with a 30% higher risk (per 50 g/day, RR = 1.30, 95% CI = 1.17 to 1.45). Matching the serving sizes, each 100 g/day of processed meats was associated with an approximately 70% higher risk (RR = 1.69, 95% CI = 1.37 to 2.10). Translated to weekly servings, each 100 g/week of unprocessed red meats had no significant association with CVD mortality (RR = 1.01, 95% CI = 1.00, 1.02), and each 100 g/week of processed meats was associated with 8% higher risk (RR = 1.08, 95% CI = 1.05, 1.11).

Do these findings suggest cause and effect? Observational studies can be limited by residual confounding, that is, the observed associations being due to other unmeasured or poorly measured factors. However, when considering such effects, it is crucial to consider plausible directions of confounding. As seen in previous studies, unprocessed and processed meat consumption in EPIC were each associated with higher-risk demographics and worse lifestyles, including older age, higher body mass index lower fruit intake, greater current smoking, and lower education; conversely, many of these associations were attenuated or reversed for poultry consumption. Although the authors adjusted for these factors, residual confounding could still be present as a result of imperfect covariate measurement. In addition, the authors did not adjust for other key dietary confounders such as fiber, whole grains, nuts, legumes, fish, and *trans* fats. Based on the associations of meat intake with these risk factors, residual confounding could overestimate the harmful associations of processed meat consumption and the protective associations of poultry consumption. However, residual confounding could not plausibly explain the absence of a link between unprocessed red meats and CVD, as the direction of residual bias in this case would be toward showing more harm, not less.

A second method to evaluate potential confounding is use of a ‘negative control’, that is, a health outcome on which the risk factor of interest would have little plausible effect [11]. In the EPIC investigation, when other causes of death were evaluated, intake of unprocessed red meat was not associated with cancer, digestive, respiratory, or other deaths, whereas intake of processed meat was associated with higher rates of cancer and other deaths (with a smaller magnitude than for CVD deaths) and was not associated with respiratory or digestive deaths. The absence of associations of processed meat intake with biologically unrelated causes of death supports a low likelihood of confounding as an explanation for the observed higher risks of CVD and cancer deaths.

What are the implications of these findings? In 2010, we performed a meta-analysis of observational studies that showed no significant association between intake of unprocessed red meat and coronary heart disease (CHD) (per 100 g/day, RR = 1.00, 95% CI = 0.81 to 1.23), and significant positive associations between processed meat intake and CHD (per 50 g/day, RR = 1.42, 95% CI = 1.07 to 1.89) [5]. However, whereas the findings for processed meat were based on 21,308 incident CHD events, the studies available for our meta-analysis of unprocessed red meats and CHD covered less than 1,000 cases. Subsequent analyses from large prospective cohorts in the USA supported stronger associations of processed meat intake with CVD, but also suggested statistically significant, although modest, associations of unprocessed red meats [12,13]. This investigation in EPIC, including nearly half a million participants across 10 European countries and more than 5,000 cardiovascular events, confirms that consumption of processed meat is strongly associated with CVD risk, and that consumption of unprocessed red meat has little to no association.

These findings, taken together with previous studies, have important implications for understanding how meat consumption influences cardiovascular health. In previous analyses, we found that average contents of saturated fat, cholesterol, and heme iron are similar between unprocessed red meats and processed meats (indeed, average cholesterol and heme iron contents are lower in processed meats) [5]. The strong association of processed meats with CVD, compared with the weak or absent association of unprocessed red meats with CVD, suggests that none of these ingredients have major effects on CVD risk. This is supported by evidence for no overall association of saturated-fat consumption with incident CHD [2-4], and little overall association of dietary cholesterol with CHD [14].

These findings also inform the extent to which other meat ingredients might be relevant for risk. Experimental evidence suggests that trimethylamine N-oxide, a metabolite of L-carnitine formed by intestinal microbiota, is pro-atherogenic [9], yet, unprocessed red meats, which have the highest L-carnitine content, have little association with CHD, whereas processed meats, which are commonly made from pork or even poultry that contains much lower L-carnitine levels, are associated with higher CHD risk. In sum, these results suggest that trimethylamine N-oxide may not mediate the observed associations with risk.

Preservatives are the most notable difference between unprocessed and processed meats. In the USA, processed meats contain an average of 400% more sodium and 50% more nitrates than unprocessed red meats [5]. The predicted blood-pressure effects of the high sodium content alone can account for more than 2/3 of the observed relationship between processed meats and CHD risk [15].

## Conclusion

The global pandemics of CVD, diabetes, cancers, and obesity have dramatically increased the interest of the public, policy-makers, media, and food industry in how dietary habits influence health and disease. Thus, reports such as those by Rohrmann and colleagues [10] are crucial for both informing science and educating the public. A growing literature provides compelling evidence that intake of processed meat increases CVD risk, whereas intake of unprocessed red meat has a relatively small or no effect. Yet, rather than focusing on preservatives and processing, many dietary guidelines continue to emphasize eating lean (lower-fat) meats. The food industry has taken up this call, heavily promoting low-fat processed meats. Restaurant and fast-food chains that promote low-fat deli meat sandwiches are notable offenders, promoting sandwiches containing highly processed meats, refined grains, and processed cheese as a ‘healthy’ choice because they are ‘low fat.’ Few meals could be worse for health. Public-health guidance should prioritize avoidance of processed meats, whether red or white, or lower-fat or higher-fat. Furthermore, given the likely contribution of sodium in the harmful health effects, the food industry should substantially reduce sodium and other preservatives in processed meats. In addition, although consumption of unprocessed red meat appears to be relatively neutral for CVD risk, no evidence suggests cardiovascular benefits, and many healthier dietary choices are available, such as fish, nuts, and legumes. Cattle farming also induces devastating environmental effects, dramatically increasing greenhouse-gas production, water wastage, and deforestation [16]. Health effects in humans aside, red-meat consumption is clearly bad for the health of our planet. Dietary recommendations should continue to move away from fat-based guidelines and instead focus upon foods and dietary patterns, including increased consumption of fruits, vegetables, nuts, whole grains, and fish, and avoidance of processed meats, other high-sodium foods, partially hydrogenated vegetable oils, and refined grains, starches, and sugars.

## Abbreviations

CHD: Coronary heart disease; CVD: Cardiovascular disease; EPIC: European Investigation into Cancer; RR: Relative risk.

## Competing interest

DM has received modest *ad hoc* consulting fees for scientific presentations from Foodminds and Nutrition Impact. The other authors have no conflicts of interest to declare.

## Authors’ contributions

RM and DM conceived of the study, drafted the manuscript, and critically revised the content. GM and ML critically revised the content. All authors read and approved the final manuscript.

## Authors’ information

DM is a cardiologist and epidemiologist whose research focuses on lifestyle, in particular diet, and global cardiometabolic health. He is co-Director of the Harvard Program in Cardiovascular Epidemiology; Associate Professor, Division of Cardiovascular Medicine, Brigham and Women’s Hospital and Harvard Medical School, and Associate Professor, Department of Epidemiology, Harvard School of Public Health.

ML is physician and epidemiologist interested in diet, lifestyle and cardiometabolic disease. He is the associate director of ESMaestras (Mexican Teachers' Cohort) at the National Institute of Public Health (Mexico); Research Fellow, Department of Epidemiology, Harvard School of Public Health; and Associate Researcher, E3N cohort in France.

RM is a clinical dietician and epidemiologist who specializes in nutritional and cardiovascular epidemiology, with a focus on diet and global chronic disease. She is the Director of the 1^st^ Hellenic National Health and Nutrition Examination Survey (HNHANES); Research Associate, Department of Food Science and Human Nutrition, Agricultural University of Athens; and Research Associate, Department of Epidemiology, Harvard School of Public Health. GM is an MD PhD, the Medical Director of the 1^st^ HNHANES; and Research Associate, Department of Food Science and Human Nutrition, Agricultural University of Athens.

## Pre-publication history

The pre-publication history for this paper can be accessed here:

http://www.biomedcentral.com/1741-7015/11/136/prepub

## References

[B1] US Department of Agriculture Dietary Guidelines For Americans 2010 http://www.cnpp.usda.gov/Publications/DietaryGuidelines/2010/PolicyDoc/PolicyDoc.pdf

[B2] Siri-Tarino PW Sun Q Hu FB Krauss RM Meta-analysis of prospective cohort studies evaluating the association of saturated fat with cardiovascular disease Am J Clin Nutr 2010 91 535 546 10.3945/ajcn.2009.27725 20071648 PMC2824152

[B3] Mente A de Koning L Shannon HS Anand SS A systematic review of the evidence supporting a causal link between dietary factors and coronary heart disease Arch Intern Med 2009 169 659 669 10.1001/archinternmed.2009.38 19364995

[B4] Micha R Mozaffarian D Saturated fat and cardiometabolic risk factors, coronary heart disease, stroke, and diabetes: a fresh look at the evidence Lipids 2010 45 893 905 10.1007/s11745-010-3393-4 20354806 PMC2950931

[B5] Micha R Wallace SK Mozaffarian D Red and processed meat consumption and risk of incident coronary heart disease, stroke, and diabetes mellitus: a systematic review and meta-analysis Circulation 2010 121 2271 2283 10.1161/CIRCULATIONAHA.109.924977 20479151 PMC2885952

[B6] Rajpathak S Ma J Manson J Willett WC Hu FB Iron intake and the risk of type 2 diabetes in women: a prospective cohort study Diabetes Care 2006 29 1370 1376 10.2337/dc06-0119 16732023

[B7] Lee DH Folsom AR Jacobs DR Jr Dietary iron intake and Type 2 diabetes incidence in postmenopausal women: the Iowa Women's Health Study Diabetologia 2004 47 185 194 10.1007/s00125-003-1307-1 14712349

[B8] Zhao Z Li S Liu G Yan F Ma X Huang Z Tian H Body iron stores and heme-iron intake in relation to risk of type 2 diabetes: a systematic review and meta-analysis PLoS One 2012 7 e41641 10.1371/journal.pone.0041641 22848554 PMC3406072

[B9] Koeth RA Wang Z Levison BS Buffa JA Org E Sheehy BT Britt EB Fu X Wu Y Li L Intestinal microbiota metabolism of l-carnitine, a nutrient in red meat, promotes atherosclerosis Nat Med 2013 19 576 585 10.1038/nm.3145 23563705 PMC3650111

[B10] Rohrmann S Overvad K Bueno-de-Mesquita HB Jakobsen MU Egeberg R Tjonneland A Nailler L Boutron-Ruault MC Clavel-Chapelon F Krogh V Meat consumption and mortality - results from the European Prospective Investigation into Cancer and Nutrition BMC Med 2013 11 63 10.1186/1741-7015-11-63 23497300 PMC3599112

[B11] Lipsitch M Tchetgen Tchetgen E Cohen T Negative controls: a tool for detecting confounding and bias in observational studies Epidemiology (Cambridge, Mass) 2010 21 383 388 10.1097/EDE.0b013e3181d61eeb PMC3053408 20335814

[B12] Bernstein AM Sun Q Hu FB Stampfer MJ Manson JE Willett WC Major dietary protein sources and risk of coronary heart disease in women Circulation 2010 122 876 883 10.1161/CIRCULATIONAHA.109.915165 20713902 PMC2946797

[B13] Pan A Sun Q Bernstein AM Schulze MB Manson JE Stampfer MJ Willett WC Hu FB Red meat consumption and mortality: results from 2 prospective cohort studies Arch Intern Med 2012 172 555 563 10.1001/archinternmed.2011.2287 22412075 PMC3712342

[B14] Rong Y Chen L Zhu T Song Y Yu M Shan Z Sands A Hu FB Liu L Egg consumption and risk of coronary heart disease and stroke: dose–response meta-analysis of prospective cohort studies BMJ 2013 346 e8539 10.1136/bmj.e8539 23295181 PMC3538567

[B15] Micha R Michas G Mozaffarian D Unprocessed red and processed meats and risk of coronary artery disease and type 2 diabetes–an updated review of the evidence Curr Atheroscler Rep 2012 14 515 524 10.1007/s11883-012-0282-8 23001745 PMC3483430

[B16] Steinfeld H Gerber P Wassenaar T Vinvent C Rosales M de Haan C Livestock's Long Shadow: Environmental Issues and Options 2006 Rome: FAO

